# Oral Health Status of Children and Adolescents Living with HIV Undergoing Antiretroviral Therapy: A Systematic Review and Meta-Analysis

**DOI:** 10.3390/ijerph191912864

**Published:** 2022-10-08

**Authors:** Phoebe Pui Ying Lam, Ni Zhou, Hai Ming Wong, Cynthia Kar Yung Yiu

**Affiliations:** 1 Paediatric Dentistry, Faculty of Dentistry, the University of Hong Kong, Pokfulam, Hong Kong SAR, China; 2 School of Stomatology, Kunming Medical University, Kunming 650032, China

**Keywords:** HIV, antiretroviral therapy, oral health, children, systematic review, meta-analysis

## Abstract

Antiretroviral therapy (ART) increases the survival of HIV-infected children, but might also bring in oral health-related side effects and increase their risks of oral diseases. The review compared the oral health status of children living with HIV (CLWH) undergoing ART with healthy controls. Dual independent screening and study selection from four electronic databases and manual searches, data extraction, risk of bias assessment, and quality-of-evidence evaluation with Grading of Recommendations Assessment Development and Evaluation were performed. Twelve studies were included in qualitative and quantitative analysis. CLWH taking ART had a significantly higher prevalence of periodontal diseases (OR = 3.11, 95% CI 1.62–5.97), mucosal hyperpigmentation (OR = 20.35, 95% CI 3.86–107.39), and orofacial-related opportunistic infections than healthy controls. No significant differences regarding caries prevalence and tooth development were identified. Those with CD4+ T-cell counts below 250 cells/mm^3^ were more likely to manifest opportunistic infections, while medication duration had minimal influence on the prevalence of orofacial opportunistic infections. The current findings did not identify HIV and antiretroviral status as predisposing factors to dental caries, but affirmed the associated increased risk of periodontal diseases, mucosal hyperpigmentation and candidiasis.

## 1. Introduction

With the implementation of HIV prevention campaigns and advancement in anti-retroviral therapy (ART), the new incidence of children living with HIV (CLWH) had declined significantly [1]. Over 70% of HIV-infected women could have access to effective ART during pregnancy, delivery, and breastfeeding [1]. The risk of vertical transmission of HIV has been reduced to less than 6% [1].

For CLWH, the current treatment approach involves the prescriptions of combined ART or so-called highly active antiretroviral therapy (HAART) [2]. HAART refers to a mixed prescription of ART agents, for instance, two nucleoside reverse transcriptase inhibitors (NRTI) with either a protease inhibitor (PI) or non-nucleoside reverse transcriptase inhibitor (NNRTI) [2]. With the early onset of HAART, a majority of CLWH can live an asymptomatic life and continue to thrive [3].

However, ART may bring along adverse effects to the surviving CLWH, impacting their general health and quality of life. The most commonly reported side effects include skin rashes, anemia, hepatotoxicity, and other detrimental damages to the gastrointestinal, metabolic, and renal systems [4,5]. Oral health-related side effects associated with ART have also been reported. Reduced salivary flow among patients taking protease inhibitors and didanosine [6] might predispose them to higher risks of dental caries and other oral diseases. Susceptibility to dental caries may be exacerbated due to prolonged use of sweetened liquid oral medication among pediatric patients [7]. Oral ulcerations have been identified among HIV- infected patients, potentially impeding usual oral hygiene maintenance. Nonetheless, without the control of confounders and ‘case-control’ comparisons with other typically developing individuals, definitive statements regarding the association between ART to oral health diseases still cannot be established.

ART also does not completely eradicate orofacial lesions and opportunistic infections, including oral candidiasis, mucosal hyperpigmentation, linear gingival erythema, angular cheilitis, etc. [8,9,10]. CLWH taking ART might still present with the above oral manifestations, despite a comparatively smaller incidence compared with HIV-treatment naïve patients [11]. Nevertheless, confounders including age, sociodemographic factors, duration of treatment, CD4+ T-cell counts, and other immunological parameters were not systematically evaluated in the published reviews and literature [12].

The present a systematic review aimed to compare the oral health status of CLWH and adolescents undergoing ART with healthy controls, including dental caries prevalence and severity, oral hygiene and periodontal diseases, orofacial diseases, saliva immuno-globulins, oral candidiasis, and dental development.

## 2. Materials and Methods

This systematic review was conducted following the PRISMA guidelines and was registered on PROSPERO (registration number CRD42019148245). The following PECO(S) statements were proposed:

(P)—Participants were children and adolescents below 18;

(E)—Exposure was those undergoing ART and HAART treatment; in this present review, ART refers to all the treatment regimens used to treat HIV, while HAART specifically refers to a combination of three or more antiretroviral medications [13].

(C)—Healthy controls;

(O)—Outcomes measures including:

1. Dental caries
2. Oral hygiene and periodontal health status
3. HIV-related orofacial diseases based on WHO clinical staging and immunological classification, including:
  - Stage 2 (angular cheilitis, herpes zoster, linear gingival erythema, recurrent oral ulcerations, parotid enlargement
  - Stage 3 (oral candidiasis, oral hairy leukoplakia, acute necrotizing ulcerative gingivitis/periodontitis)
  - Stage 4 (Herpes simplex infection, Kaposi sarcoma)
4. Saliva immunoglobulins quantity
5. Oral candidiasis
6. Dental development

(S)—Studies included were case-control observational studies with full text reports available in English.

### 2.1. Search Strategies

Utilizing MeSH terms and broad keywords (Appendix A), a strategic literature search was performed systematically. Four electronic databases (Ovid Embase, Ovid MEDLINE, Pubmed, and Scopus) were searched from inception to 29 July 2022. Reference lists of past relevant literature reviews were also screened to identify any additional pertinent reports.

### 2.2. Study Selection

Two reviewers (PPYL and NZ) independently assessed the eligibility of retrieved articles based on their titles and abstracts. Any disagreement was resolved by consensus or consulting the third reviewer (HMW). Cohen’s kappa coefficient (κ) was calculated to determine the agreement between reviewers.

### 2.3. Data Extraction and Management

Relevant data from included studies were extracted independently, using a standardized data extraction form. Dichotomous outcomes, including prevalences of caries, oral neoplasms, and oral bacterial, viral, and fungal infections; odds ratio (OR) and 95% confidence intervals (CI) were calculated and compared.

The mean and standard deviation were obtained for continuous outcome, including the number of decayed, missing due to decay, filled teeth, surfaces in permanent dentition (DMFT/DMFS), primary dentition (dmfs/dmfs), the mean number of untreated decayed teeth, and surfaces in permanent (DT/DS) and primary dentitions (dt/ds) [14], plaque index and gingival index [15,16], salivary flow rate and pH.

When computational problems occurred due to no events in at least one group, a fixed value (0.5) was added to each cell in the 2 × 2 tables. Adjusted OR was calculated and assessed with zero-cell corrections [17].

### 2.4. Subgroup Analyses

Subgroup analyses were conducted to separately compare different outcomes in control of confounders, including different dentitions, CD4+ T-cell counts, and other immunological factors, durations, and types of medication, if available.

### 2.5. Risk of Bias (RoB) Assessment

RoB in non-randomized studies of interventions tool (ROBINS-I tool) was used to determine the RoB of the included observational studies [18]. Each report was evaluated in seven domains of bias, including (I) bias due to confounding, (II) bias in a selection of participants in the study, (III) bias in the classification of interventions, (IV) bias due to deviation from intended interventions, (V) bias due to missing data, (VI) bias in measurement of outcomes, and (VII) bias in selection reported. Each domain was evaluated with the guided signal questions provided, and an overall rating of low, moderate, serious, or critical RoB was determined.

### 2.6. Data Synthesis

Meta-analyses were carried out with Stata version 13.1 (StataCorp, College Station, TX, USA). A fixed-effects model was employed when there were fewer than five relevant studies; otherwise, a random-effect model was adopted [19]. Only narrative presentation of results without meta-analyses if results were substantially heterogeneous or significantly divergent. In the presence of studies with low validity, sensitivity analyses were performed to verify whether the effect estimate was significantly affected [19].

### 2.7. Assessment of Heterogeneity

Both I^2^ statistics and Chi Square tests were employed to assess heterogeneity. Heterogeneity was considered as substantial when I^2^ > 50% and *p* < 0.10 in Chi Square test [19].

### 2.8. Assessment of Publication Bias

Funnel plots were used for the assessment when the outcome effect estimates were obtained from over 10 studies [20].

### 2.9. Assessment of Quality of Evidence

The quality of evidence for all outcomes was evaluated following the GRADE approach [21]. Beginning as low quality of evidence as only observational studies were included, the reviewers might consider further downgrading when serious issues related to RoB, imprecision, inconsistency, indirectness, and publication bias were identified.

## 3. Results

### 3.1. Study Selection

**Figure 1:**
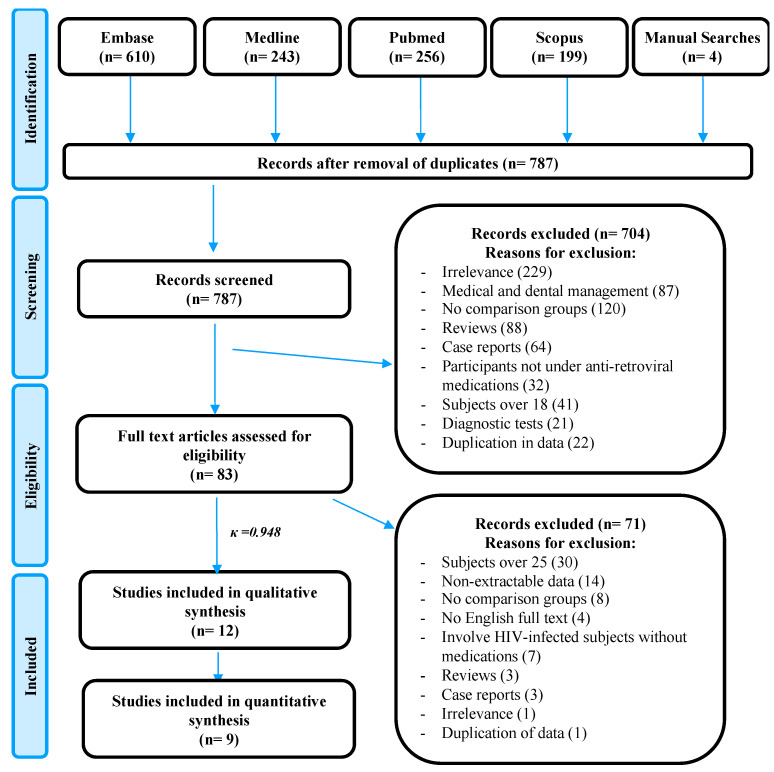
PRISMA flowchart of the current review.

A systematic search in the literature yielded 787 reports after duplicate removal. Based on titles, keywords, and abstracts, 83 articles were retrieved for a full text reading. Final qualitative and quantitative syntheses included 12 case-control studies (Kappa = 0.948), whereas 71 articles were excluded (Figure 1).

### 3.2. Study Characteristics

The 12 included studies were conducted in Brazil, India, Saudi Arabia, and West Africa [9,22,23,24,25,26,27,28,29,30,31,32]. One-thousand-and-two HIV-infected subjects were recruited from hospitals, AIDS clinics, or centers; while 975 healthy-matched controls were either siblings or subjects from the nearby school and clinics (Table 1).

### 3.3. RoB

**Figure 2:**
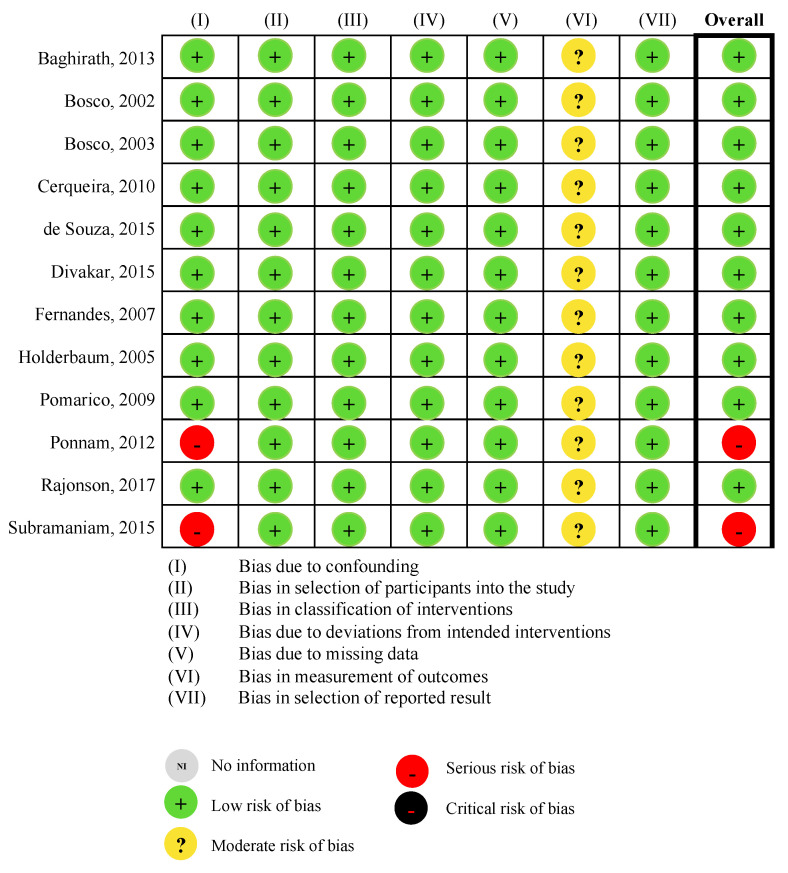
Risk of bias assessment with ROBINS-I [9,22,23,24,25,26,27,28,29,30,31,32].

The studies by Ponnam et al. (2012) and Subramaniam and Kumar (2015) were considered as of severe risk of bias due to confounding and overall RoB as no matching of socioeconomic status of controls were performed. The other ten studies were assessed as of low risk of overall RoB, Figure 2.

### 3.4. Dental Caries Experience

**Figure 3:**
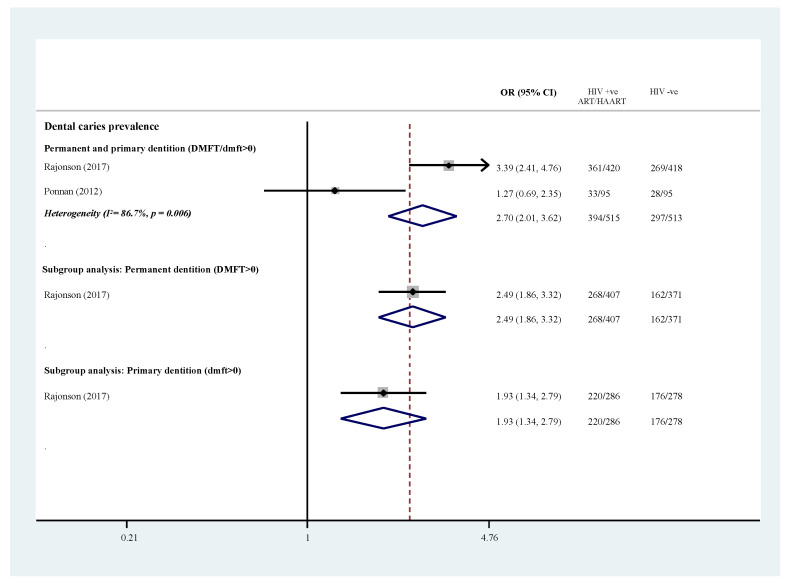
Prevalence of dental caries: HIV-infected individuals under antiretroviral. medications versus healthy controls [9,31].

Two studies with 1028 case-control subjects contributed to the outcomes regarding dental caries prevalence (DMFT/dmft/DMFS/dmfs > 0). Ponnam et al. (2012) identified no significant differences (OR = 1.27, 95% CI 0.69–2.35; *p* > 0.05)]; while Rajonson et al. (2017) noticed significantly higher caries prevalence among the CLWH under ART in both dentition (primary dentition: OR = 2.49, 95% CI 1.86–3.32, *p* < 0.001; permanent dentition: OR = 1.93, 95% CI 1.34–2.79; *p* < 0.001), Table 2 and Figure 3.

Rajonson et al. (2017) reported that CLWH also had a significantly higher median DMFT/dmft/DMFS/dmfs than HIV-uninfected children. Using a zero-inflated negative binomial regression model to evaluate potential confounders, HIV status was found to be associated with both higher caries prevalence and median DMFT/dmft/DMFS/dmfs among children below 12 years old; whereas only significantly higher caries prevalence but not median DMFT/dmft/DMFS/dmfs was found for children above 12 years.

### 3.5. Oral Hygiene and Periodontal Status

For oral hygiene status, only one eligible study [31] compared and reported the simplified oral hygiene index of CLWH with healthy controls, where no significant differences were found between the two groups.

**Figure 4:**
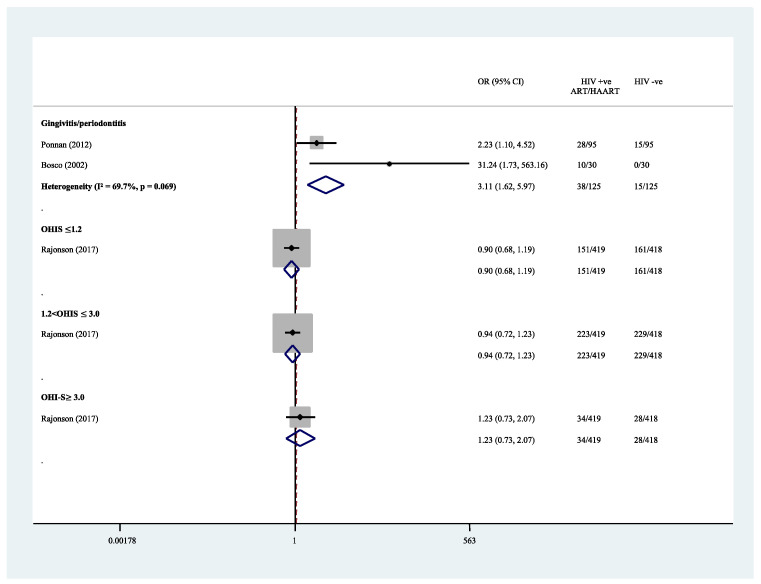
Prevalence of periodontal diseases: HIV-infected individuals under antiretroviral. medications versus healthy controls [9,23,31].

For periodontal diseases, Bosco and Birman (2002) and Ponnam et al. (2012), with 250 case-control subjects, compared the prevalence of periodontal diseases between CLWH and healthy controls. Both studies reported significantly higher prevalence of periodontal diseases among CLWH and adolescents with ART. However, both studies did not specify the indices they used in determining gingivitis and periodontitis. Bosco and Birman (2002) further grouped the CLWH based on their CD4+ T-cell counts and degree of suppression and found gingivitis only prevailed in children with moderate to severe suppression (below 999 cells/mm^3^). Despite consistent findings, moderate heterogeneity is shown (OR = 3.11, 95% CI 1.62–5.97, *p* = 0.001; I^2^ = 69.7%, *p* = 0.069) in Table 2 and Figure 4.

### 3.6. HIV-Related Orofacial Diseases

**Figure 5:**
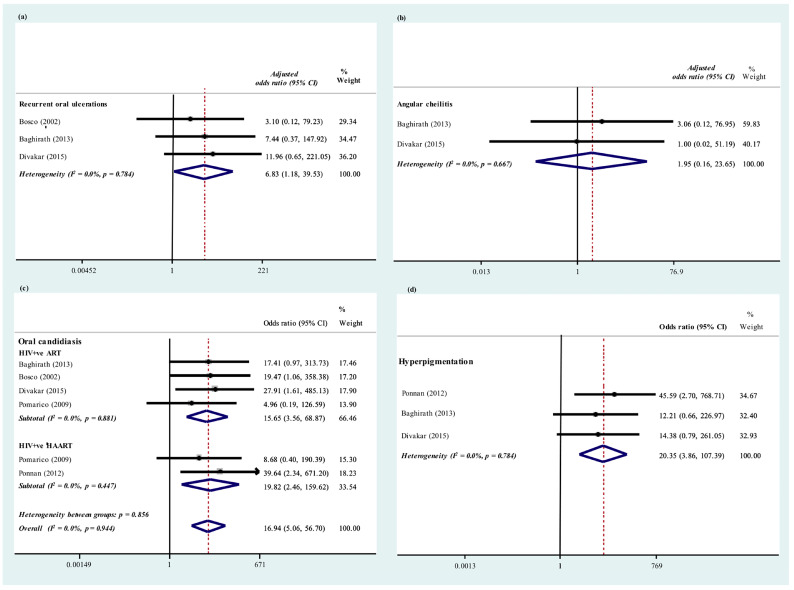
Prevalence of (**a**) recurrent oral ulceration (**b**) angular cheilitis (**c**) oral candidiasis (**d**) hyperpigmentation: HIV-infected individuals under antiretroviral medications versus healthy controls [9,22,23,27,30].

**Figure 6:**
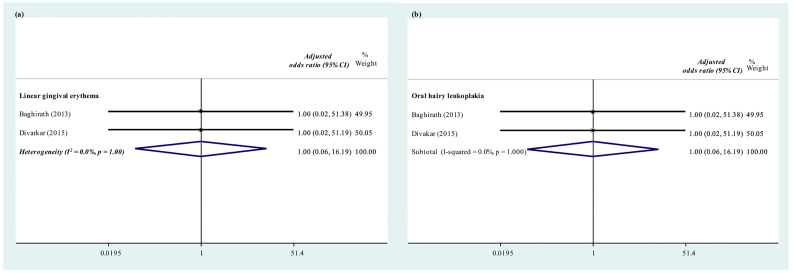
Prevalence of (**a**) linear gingival erythema (**b**) oral hairy leukoplakia: HIV-infected individuals under antiretroviral medications versus healthy controls [22,27].

HIV-related orofacial manifestations including recurrent oral ulcerations, oral candidiasis, ulcerative stomatitis, hyperpigmentation, and parotid enlargement were not found among healthy individuals. The adjusted OR of recurrent oral ulceration (OR = 6.83; 95% CI 1.18–39.53; *p* < 0.001; I^2^ = 0.0%, *p* = 0.784), oral candidiasis (OR = 16.94; 95% CI 5.06–56.70; *p* < 0.001; I^2^ = 0.0%, *p* = 0.944) and hyperpigmentation (OR = 20.35; 95% CI 3.86–107.39; *p* < 0.001; I^2^ = 0.0%, *p* = 0.784) were reported in Table 2 and Figure 5. However, the meta-analysis suggested that angular cheilitis may still be found among healthy individuals and the prevalence of angular cheilitis of the two groups did not significantly differ from each other (OR = 1.95; 95% CI 0.16–23.65; *p* < 0.001; I^2^ = 0.0%, *p* = 0.667). Manifestations including oral hairy leukoplakia and linear gingival erythema were not found in either CLWH or healthy controls, Figure 6.

### 3.7. Saliva Immunoglobulins Quantity

One study compared and reported the salivary IgA (SIgA) concentration [30]. There was no significant difference in the total SIgA among HIV-ART, HIV-HAART children, and healthy controls, but there was a significantly higher concentration of anti-C albicans SIgA in the former two groups.

### 3.8. Oral Candidiasis

Five studies compared the prevalence of oral candidiasis infection between HIV-infected individuals undergoing medications with healthy individuals [9,22,23,27,30]. Consistent findings suggested that significantly higher proportion of children with HIV still present with opportunistic infections like oral candidiasis although they were already undergoing antiviral medications (OR = 16.94, 95% CI 5.06–56.70; *p* < 0.001; I^2^ = 0.0%, *p* = 0.944), Table 2.

### 3.9. Dental Development

Three studies [26,28,29] used either Nolla’s method or Willems’ method to assess dental age. The age of the included subjects in the three studies ranged from 4–16 years old, with even distributions of males and females. Fernandes et al. (2007) and de Souza et al. (2015) reported no significant difference in dental development between the two groups.

Holderbaum et al. (2005) reported that the mean dental age assessed by Nolla’s method of HIV-infected males had no significant difference with the chronological age, while healthy male controls had their chronological age significantly overestimated. Whereas the dental age of HIV-infected females obtained with Nolla’s method was significantly lower than their chronological age, however, the same underestimation of chronological age was noticed in the control group.

### 3.10. Other Associated Factors

Meta-regression could not be conducted due to the lack of raw data. Other confounding factors including CD4+ T-cell counts and duration of the medications were hence evaluated separately.

Baghirath (2013) and Divakar (2015) reported that CLWH having CD4+ T-cell counts below 250 cells/mm^3^ were nearly two times as likely to have HIV-related oral lesions (OR = 1.916, 95% CI 0.613–2.513, *p* = 0.001; OR = 1.999, 95% CI 0.129–3.688, *p* = 0.001 respectively).

Two studies [22,27] evaluated and compared the effect of the duration of HAART on the prevalence of opportunistic infections. The opportunistic infections investigated include linear gingival erythema, hairy leukoplakia, angular cheilitis, and oral ulcers. Except for candidiasis (OR = 17.93, 95% CI 3.69–83.12, *p* = 0.002); no significant differences in the prevalence of other opportunistic infection manifestations were found between those taking ART below three years and those taking it three years or more. However, fewer children who had taken ART not less than three years had CD4+ T-cell counts below 250 cells/mm^3^ (Table 2).

## 4. Discussion

CLWH had been reported to have poorer oral health status [33]. However, conclusions were mostly drawn from cross-sectional studies without head-to-head comparisons with their healthy counterparts. Most cross-sectional studies were conducted in developing countries, the increased caries prevalence and severity might be contributed by other confounders; for instance, socioeconomic status and water fluoridation.

Divergent results were found when comparing the caries experience between HIV-ART/HAART and healthy individuals, with one study showing higher caries prevalence among HIV-ART/HAART individuals [9] while the other found no significant difference [31]. The effect estimate was provided by 1–2 small-scale studies with moderate to high RoB. Due to serious risks of bias, inconsistencies, or imprecisions, the certainties of evidence were severely compromised, precluding valid conclusions to be drawn in Supplementary Material Table S1. The current evidence does not suggest CLWH under medical management are more prone to dental caries and the disease itself is no predisposing factor to caries. Other risk factors, for instance, inadequate oral hygiene and cariogenic diet, might play a more fundamental role in the disease progression. More high-quality case-control studies are warranted to determine if CLWH are more susceptible to dental caries.

Consistent results demonstrated that HIV-ART/HAART individuals were more likely to have mucosal hyperpigmentation. ART was associated with elevated production of á-melanocyte-stimulating hormone(á-MSH), which might be responsible for the increased melanin production and coincided with the current findings [34]. Nonetheless, the treatment duration seems to have minimal influence on hyperpigmentation based on the pooled data of two small-scale studies available. Other than being drug-induced, oral hyperpigmentation was also found in other CLWH without taking any medications. These idiopathic lesions appeared as brown-black macules with well-defined margins, and melanin was only identified in keratinocytes of the basal cell layer or in extracellular foci [34]. One proposed theory was that the melanocytes were stimulated during HIV infection, resulting in immunopathologic changes in the oral mucosa [35], but further validations of such theories remain scarce. Further research is warranted due to the low certainty of the evidence.

Inconclusive results were also found on whether ART treatment would affect dental mineralization [26,28,29]. Dental maturity or dental age has been a debatable method to assess chronological age, as dental development markedly varies across different ethnic groups. Additionally, inaccuracies and overestimation of chronological age by Nolla and Willems method had been reported in the literature, ranging from 0.05 to 1.15 years and 0.06 to 0.26 years respectively [36,37]. The reported errors between dental age and chronological might not be because of the medications, but the errors of the assessment method itself.

It is not surprising to find that opportunistic infections were more prevalent among HIV-infected individuals when compared with healthy controls. The most common and distinctive opportunistic infections which were only found among HIV-infected individuals were oral candidiasis. Other opportunistic infections, such as linear gingival erythema, oral hairy leukoplakia, and angular cheilitis were not common in both healthy and HIV-infected individuals. The occurrence of oral candidiasis was more likely to be associated with the drop in CD4+ T-cell counts. Moreover, children with the presence of oral candidiasis were five times more likely to experience dental caries than those without [38], which might be contributed to the capability of C. albicans to acclimate and thrive in a wide range of pH [39], especially in acidic environment created by the cariogenic biofilm. Frequent screening and detection of oral candidiasis might be necessary to assess the disease status of HIV and the treatment progress.

This review was conducted following PRISMA guidelines [40]. The strengths include the use of both qualitative and quantitative synthesis, sensitivity analyses, and comprehensive evaluation of quality evidence using the GRADE approach [18].

The limitations include the inevitable exclusion of non-English translated reports, exclusion of subjects over 25, or non-extractable data. Due to small sample sizes and limited number of relevant studies, the difficulty in detecting heterogeneity increased. Although a higher *p*-value of 0.1 instead of the usual 0.05 was used for each meta-analysis, it might still not be sensitive enough given the clinical, methodological, and statistical diversity of the included studies [19]. Meanwhile, publication bias and meta-regression could not be conducted due to limited studies and the inability to pull relevant data from reports respectively. For future research, more high-quality case-control studies with matched controls controlling for sociodemographic and other confounding factors are warranted to provide information about the oral health status of CLWH undergoing ART.

## 5. Conclusions

The present review identified a significantly higher prevalence of periodontal diseases, hyperpigmentation, oral candidiasis, and ulcerative stomatitis among CLWH undergoing ART compared with healthy controls. However, no significant difference regarding caries prevalence and severity, oral hygiene status, saliva immunoglobulins, and dental development was found between CLWH and adolescents undergoing ART with healthy controls. The underlying factors for opportunistic infections might be attributed to the drop of CD4+ T-cell counts below 250 cells/mm^3^. However, due to the dearth of high-quality, case-control studies, more definitive statements regarding the oral health status of CLWH undergoing ART cannot be established.

## Figures and Tables

**Table 1:** Characteristics of included studies.

	Study (Year, Country ^†^) ^P/R^	Number of Subjects (% Males)		Age Range (Year)	Recruitment	Inclusion (I) /Exclusion (E) Criteria ^‡^	Confounders Evaluated	Outcome Measures
1	Baghirath [22] (2013; IND) ^P^	HIV+ve ART	50 (46)	5–12	Nireekshana ART centre, Hyderabad (NGO)	(I) Seropositive for antibody to HIV when tested by a particle agglutination test for antibodies to HIV and enzyme-linked immunosorbent assay (ELISA) (E) HIV-infected subjects with a history of local radiation therapy to the head and neck region.	✓ Duration of therapy	✓ Linear gingival erythema ✓ Hairy leukoplakia ✓ Angular cheilitis ✓ Oral ulcers ✓ Candidiasis ✓ Hyperpigmentation
		HIV+ve ART	50 (50)	5–12	Nearby school	(I) Systemically healthy subjects (children with a negative history of anaemia, oral ulceration, herpetic lesions, etc.)		
2	Bosco [23] (2002, BRA) ^P^	HIV+ve ART	30 (NR)	2–6	Pediatric Infectology Center	(I) Born from HIV-positive mothers, presenting with anti-HIV serum, positively detected by means of ELISA and Western Blot tests after 15 months of age (I) Examined for CD4+ counts once, two to three months before clinical examination (I) Symptomatic children with AIDS, under constant and regular treatment with drugs	✓ CD4+ counts	✓ Lymphadenopathy ✓ Gingivitis ✓ Candidiasis ✓ Parotid enlargement ✓ Ulcerations
		HIV-ve	30 (NR)	2–6				
3	Bosco [24] (2003, BRA) ^P^	HIV+ve ART	30 (NR)	2–6	Pediatric Infectology Center	(I) Born from HIV-positive mothers, presenting with anti-HIV serum, positively detected by means of ELISA and Western Blot tests after 15 months of age. (I) Examined for CD4+ counts once, two to three months before clinical examination. (I) Symptomatic children with AIDS, under constant and regular treatment with drugs	✓ CD4+ counts	✓ Candidiasis
		HIV-ve	30 (NR)	2–6				
4	Cerqueira [25] (2010; BRA) ^P^	HIV+ve HAART	65 (41.5)	2–13	Pediatric AIDS Outpatients Clinic at the Federal University	(I) Definitive diagnosis for HIV infection confirmed by 2 positive ELISA tests and 1 positive Western Blot (E) Presence of fixed or removable orthodontic appliances and systemic or local antifungal treatment within the last three months	✓ Nil	✓ Dental caries prevalence and severity ✓ Candida species distribution ✓ Gingivitis ✓ Parotid enlargement ✓ Herpes infection ✓ Aphthous ulcers
		HIV-ve	40 (50)	5–18	Age and gender-matched siblings	(I) HIV-seronegative children confirmed by the aforementioned tests (E) Presence of fixed or removable orthodontic appliances and systemic or local antifungal treatment within the last three months		
5	de Souza [26] (2015, BRA) ^P^	HIV+ve HAART	80 (40)	4–15	University stomatology department	(I) Vertically infected children, with known gender; date of birth; and date of image acquisition	✓ Nil	✓ Dental mineralization chronology
		HIV-ve	80 (40)	4–15				
6	Divakar [27] (2015; SAU) ^P^	HIV+ve ART	62 (59.6)	5–15	District Hospital ART centre	(I) Positive on particle agglutination test for antibodies to HIV and enzyme-inked immunosorbent assay (ELISA) (E) History of adverse habits like tobacco, betel nut	✓ Duration of therapy	✓ Linear gingival erythema ✓ Hairy leukoplakia ✓ Angular cheilitis ✓ Oral ulcers ✓ Candidiasis ✓ Hyperpigmentation
		HIV-ve	62 (58.1)	5–15	Nearby school	(I) Systemically healthy subjects (E) History of adverse habits like tobacco, betel nut		
7	Fernandes [28] (2007; BRA)^P^	HIV-ve	50 (48)	4–13	Hospital Pediatric AIDS Service	(E) Less than four years old because of the difficulty in obtaining a panoramic radiograph (E) Infected with HIV and hospitalized because of their debilitated physical condition (E) Presented with systemic illnesses were excluded from the control group.	✓ Nil	✓ Dental mineralization chronology
		HIV+ve ART	30(56.7)	5–16	Pediatric Dentistry and Clinic for Children and Adolescents			
8	Holderbaum [29] (2005, BRA) ^P^	HIV+ve ART	30(56.7)	5–16	University Pediatric Service of the Clinical Hospital	(I) Vertically infected and were under outpatient treatment	✓ Nil	✓ Dental mineralization chronology
		HIV-ve	30 (56.7)	5–16	University School of Dentistry	(I) Did not present any other systemic condition (I) Presented chronological ages similar to those of the HIV-positive children in both phases		
9	Pomarico [30] (2009; BRA) ^P^	HIV+ve HAART	65 (41.5)	2–13	Pediatric AIDS Outpatients Clinic at the Federal University	(I) Definitive diagnosis for HIV infection confirmed by 2 positive ELISA tests and 1 positive Western Blot (E) Presence of fixed or removable orthodontic appliances and systemic or local antifungal treatment within the last three months	✓ AIDS status	✓ Candida species ✓ Specific SIgA
		HIV-ve	40 (50)	5–18	Age and gender-matched siblings	(I) HIV-seronegative children confirmed by the aforementioned tests (E) Presence of fixed or removable orthodontic appliances and systemic or local antifungal treatment within the last three months		
10	Ponnam [9] (2012, IND) ^P^	HIV+ve HAART	95 (45.3)	5–15	Anti-Retroviral Therapy (ART) Center in Government General Hospital		✓ Nil	✓ Candidiasis ✓ Caries ✓ Gingivitis/periodontitis ✓ Dental ✓ Ulcerative stomatitis ✓ Hyperpigmentation ✓ Mucocele
		HIV-ve	95 (NR)	5–15	Anti-Retroviral Therapy (ART) Center in Government General Hospital	(I) Same age group and same socioeconomic status		
11	Rajonson [31] (2016, CIV, MIL, SEN) ^P^	HIV+ve HAART	420 (53.1)	5–15	Five HIV pediatric clinics participating in International Epidemiologic Databases to evaluate AIDS (IeDEA) West Africa Consortium	(I) HIV-infected children on ART	✓ Nil	✓ Dental prevalence and severity ✓ Oral hygiene and periodontal status
		HIV-ve	418 (52.9)	5–15	Age and gender-matched siblings			
12	Subramaniam [32] (2015; IND) ^P^	HIV+ve Post-ART	25 (58.5)	6–8	HIV centers	(I) HIV infected children aged 6–8 years (I) Children prior to onset of anti-retroviral therapy and Group 2 consisting of children undergoing anti-retroviral therapy for more than 3 years	✓ Nil	✓ Specific SIgA
		HIV-ve	50	6–8	Similar geographic surroundings			

Legend of table: ^†^ ISO alpha-3 codes of Countries.^‡^ Extracted and quoted from the included articles. ^P^ Prospective study; ^R^ Retrospective study. Note. ART = antiretroviral medication; HAART = Highly Active Anti Retroviral Therapy; NR = Not reported.

**Table 2:** Comparison of HIV-infected individuals under antiretroviral medications versus healthy control.

	Study (Year)		Number of Subjects			Outcomes		
	Exposure		Control	Exposure	Control		OR	95% CI
	Dental caries prevalence and severity							
1	Ponnam (2012) [9]	HIV+ve HAART	HIV-ve	95	95	DMFT/dmft > 0	1.27	(0.69, 2.35) ^NS^
2	Rajonson (2017) [31]	HIV+ve ART	HIV-ve	420	418	DMFT/dmf t> 0	3.39	(2.41, 4.76) *↑
	Overall						2.70	(2.01, 3.62) *↑
	Rajonson (2017) [31]			407	317	DMFT > 0	2.49	(1.86, 3.32) *↑
	Rajonson (2017) [31]			286	278	dmft > 0	1.93	(1.34, 2.79) *↑
	Oral hygiene and periodontal status							
1	Bosco (2002) [23]	HIV+ve ART	HIV-ve	30	30	Gingivitis/ periodontitis	31.24	(1.73, 563.16) *↑
2	Ponnam (2012) [9]	HIV+ve HAART	HIV-ve	95	95		2.23	(1.10, 4.52) *↑
	Overall						3.11	(1.62, 5.97) ^*^↑
	Rajonson (2017) [31]	HIV+ve HAART	HIV-ve	419	418	Poor oral hygiene (OHI-S > 3)	1.23	(0.73, 2.07) ^NS^
	Oral health-related WHO clinical staging 2							
1	Baghirath (2013) [22]	HIV+ve ART	HIV-ve	50	50	Angular cheilitis ^†^	3.96	(0.12 ,76.95) ^NS^
2	Divarkar (2015) [27]			62	62		1.00	(0.02, 51.19) ^NS^
	Overall						1.95	(0.16, 23.65) ^NS^
1	Baghirath (2013) [22]	HIV+ve ART	HIV-ve	50	50	Linear gingival erythema ^†^	1.00	(0.02, 51.38) ^NS^
2	Divarkar (2015) [27]			62	62		1.00	(0.02, 51.19) ^NS^
	Overall						1.00	(0.06, 16.19) ^NS^
1	Baghirath (2013) [22]	HIV+ve ART	HIV-ve	30	30	Recurrent oral ulcerations ^†^	3.10	(0.12, 79.23) ^NS^
2	Divarkar (2015) [27]			50	50		7.44	(0.37, 147.92) ^NS^
3	Bosco (2002) [23]			62	62		11.96	(0.65,221.05) ^NS^
	Overall						6.83	(1.18, 39.53) ^*^↑
1	Bosco (2002) [23]	HIV+ve ART	HIV-ve	30	30	Persistent parotid enlargement ^†^	10.36	(0.53, 201.45) ^NS^
	Oral-health related WHO clinical staging 3							
1	Baghirath (2013) [22]	HIV+ve ART	HIV-ve	50	50	Oral candidiasis ^†^	17.41	(0.97, 313.73) ^NS^
2	Bosco (2002) [23]			30	30		19.47	(1.06, 358.38) *↑
3	Divarkar (2015) [27]			62	62		27.91	(1.61, 485.13) *↑
4	Pomarico (2009) [30]			25	40		4.96	(0.19, 126.59) ^NS^
5	Pomarico (2009) [30]	HIV+ve HAART	HIV-ve	25	40		8.68	(0.40, 190.39) ^NS^
6	Ponnam (2012) [9]			24	40		39.64	(2.34, 671.20) *↑
	Overall						16.94	(5.06, 56.70) *↑
1	Baghirath (2013) [22]	HIV+ve ART	HIV-ve	50	50	Oral hairy leukoplakia ^†^	1.00	(0.02, 51.38) ^NS^
2	Divarkar (2015) [27]			62	55		1.00	(0.02, 51.19) ^NS^
	Overall						1.00	(0.06, 16.19) ^NS^
1	None					Acute necrotizing ulcerative gingivitis/periodonitits		
					
	Oral health-related WHO clinical staging 4							
	None					Chronic herpes simplex infection	(Nil)	(Nil)
	None					Kaposi’s sarcoma	(Nil)	(Nil)
	Other oral health-related diseases and conditions							
1	Baghirath (2013) [22]	HIV+ve ART	HIV-ve	50	50	Hyperpigmentation ^†^	45.59	(2.70, 768.71) *↑
2	Divarkar (2015) [27]	HIV+ve ART	HIV-ve	62	55		12.21	(0.66, 226.97) ^NS^
3	Ponnan (2012) [9]	HIV+ve HAART	HIV-ve	95	95		14.38	(0.79, 261.05) ^NS^
	Overall						20.35	(3.86, 107.39) *↑
1	Ponnan (2012) [9]	HIV+ve HAART	HIV-ve	95	95	Mucocele ^†^	3.03	(0.12, 75.37) ^NS^
1	Ponnan (2012) [9]	HIV+ve HAART	HIV-ve	95	95	Ulcerative stomatitis ^†^	28.59	(1.67, 490.32) *↑

Legend of table: *^↑^: Significantly higher; *^↓^: Significantly lower ^†^ Adjusted OR was calculated with a fixed value (0.5) added to each cell in the 2 × 2 tables for zero-cell corrections. Note. ART = antiretroviral medication; dmft/DMFT = decayed, missing, filled teeth for primary/permanent dentition; DMFS/dmfs = decayed, missing, filled tooth surface for primary/permanent dentition; DT = dual-therapy HAART = Highly Active Anti Retroviral Therapy; NS = Not significant; NR = Not reported; OR = odds ratio; SMD = standardized mean difference; 95% CI = 95% confidence interval.

## Data Availability

The data presented in this study are available in the supplementary materials.

## Supplementary Materials

The following supporting information can be downloaded at: https://www.mdpi.com/article/10.3390/ijerph191912864/s1, Table S1: GRADE summary of findings table for the primary outcome (caries prevention and arrest) and secondary outcome.

## Appendix A. Search Strategies

PubMed Searched on 29 July 2022 **Results: 256** (((((HIV Infections[MeSH] OR HIV[MeSH] OR “sexually transmitted diseases, viral”[MeSH] OR AIDS[MeSH] OR HIV[tw] OR HIV-1[tw] OR HIV-2[tw] OR HIV1[tw] OR HIV2[tw] OR HIVinfect*[tw] OR human immunodeficiency virus[tw] OR human immune-deficiency virus[tw] OR human immuno-deficiency virus[tw] OR human immune-deficiency virus[tw] OR ((human immun*) AND (deficiency virus[tw])) OR acquired immunodeficiency syndrome[tw] OR acquired immune-deficiency syndrome[tw] OR acquired immuno-deficiency syndrome[tw] OR acquired immune-deficiency syndrome[tw] OR ((acquired immun*) AND (deficiency syndrome[tw]))))) AND ((“Adolescent”[Mesh] OR “Child”[Mesh] OR “Infant”[Mesh] OR “pediatric”[tw] OR ”paediatric”[tw] OR “adolescent”[tw] OR “teen”[tw] OR “infant”[tw]))) AND (((((((((((("Oral Health"[MeSH]) OR "Mouth Diseases"[MeSH]) OR “Oral Hygiene”[MeSH]) OR “Dental Caries”[MeSH]) OR “Periodontal Diseases”[MeSH]) OR "Gingival Diseases"[MeSH]) OR "Tooth Injuries"[MeSH]) OR "Mouth Neoplasms”[MeSH]) OR “Dentistry”[MeSH])) OR ((((((((((((oral health[tw]) OR mouth disease*[tw]) OR oral hygiene[tw]) OR mouth hygiene[tw]) OR dental caries[tw]) OR periodontal disease*[tw]) OR gingival disease*[tw]) OR tooth injur*[tw]) OR mouth tumor[tw]) OR oral cancer[tw]) OR dentistry[tw]))))) AND (((“Antiretroviral Therapy, Highly Active”[Mesh] OR “Anti-Retroviral Agents"[Mesh] OR “antiretroviral”[tw] OR “ART”[tw] OR “medication”[tw])))
Medline (Ovid MEDLINE(R) 1946 to December Week 4 2019, Ovid MEDLINE(R) In-Process and Other Non-Indexed Citations 26 July 2019) Searched on 29 July 2022 **Results: 243** 1. exp HIV Infections/ or exp HIV/ or exp sexually transmitted diseases, viral/ or exp AIDS/ 2. (HIV or HIV-1 or HIV-2 or HIV1 or HIV2 or HIVinfect* or human immunodeficiency virus or human immune-deficiency virus or human immuno-deficiency virus or human immune-deficiency virus).tw. 3. ((human immun* and deficiency virus) or acquired immunodeficiency syndrome or acquired immune-deficiency syndrome or acquired immuno-deficiency syndrome or acquired immune-deficiency syndrome or (acquired immun* and deficiency syndrome)).tw. 4. 1 or 2 or 3 5. exp adolescent/ or exp child/ or exp infant/ 6. (pediatric or paediatric or adolescent or teen or infant).tw. 7. 5 or 6 8. exp Oral Health/ or exp Mouth Diseases/ or exp Oral Hygiene/ or exp Dental Caries/ or exp Periodontal Diseases/ or exp Gingival Diseases/ or exp Tooth Injuries/ or exp Mouth Neoplasms/ or exp Dentistry/ 9. (oral health or mouth disease* or oral hygiene or mouth hygiene or dental caries or periodontal disease* or gingival disease* or tooth injur* or mouth tumor or oral cancer or dentistry).tw. 10. 8 or 9 11. 4 and 7 and 10 12. exp Antiretroviral Therapy, Highly Active/ or exp Anti-Retroviral Agents/ 13. (antiretroviral or ART or medication).tw. 14. 12 or 13 15. 11 and 14
Embase (Embase Classic+Embase 1947 to 2018 December 28) Searched on 29 July 2022 **Results: 610** 1. exp Human immunodeficiency virus infection/ or exp Human immunodeficiency virus/ or exp sexually transmitted disease/ or exp acquired immune deficiency syndrome/ 2. (HIV or HIV-1 or HIV-2 or HIV1 or HIV2 or HIVinfect* or human immunodeficiency virus or human immune-deficiency virus or human immuno-deficiency virus or human immune-deficiency virus).tw. 3. ((human immune* and deficiency virus) or acquired immunodeficiency syndrome or acquired immune-deficiency syndrome or acquired immuno-deficiency syndrome or acquired immune-deficiency syndrome or (acquired immune* and deficiency syndrome)).tw. 4. 1 or 2 or 3 5. exp adolescent/ or exp child/ or exp infant/ 6. (pediatric or paediatric or adolescent or teen or infant).tw. 7. 5 or 6 8. exp mouth disease/ or exp mouth hygiene/ or exp dental caries/ or exp periodontal disease/ or exp gingiva disease/ or exp tooth injury/ or exp mouth tumor/ or exp dentistry/ 9. (oral health or mouth disease* or oral hygiene or mouth hygiene or dental caries or periodontal disease* or gingival disease* or tooth injury* or mouth tumor or oral cancer or dentistry).tw. 10. 8 or 9 11. 4 and 7 and 10 12. exp highly active antiretroviral therapy/or exp antiretrovirus agent/ 13. (antiretroviral or ART or medication).tw. 14. 12 or 13 15. 11 and 14
Scopus Searched on 29 Jul 2022 **Results: 199** (((TITLE-ABS-KEY (“HIV Infections” OR HIVOR “sexually transmitted diseases, viral” OR Aids OR “HIV-1” OR “HIV-2” OR HIV1 OR HIV2 OR “human immunodeficiency virus” OR “human immune-deficiency virus” OR "human immuno-deficiency virus” OR “human immune-deficiency virus”) OR TITLE-ABS-KEY (“acquired immunodeficiency syndrome” OR “acquired immune-deficiency syndrome” OR “acquired immuno-deficiency syndrome” OR “acquired immune-deficiency syndrome”))) AND (TITLE-ABS-KEY (adolescent OR child OR infant OR pediatric OR paediatric OR teen)) AND ( TITLE-ABS-KEY (“oral health” OR “Mouth Diseases” OR “Oral Hygiene” OR “Dental Caries” OR “Periodontal Diseases” OR “Gingival Diseases” OR “Tooth Injuries” OR “Mouth Neoplasms” OR “mouth tumor” OR “oral cancer” OR dentistry))) AND (TITLE-ABS-KEY (“Antiretroviral Therapy, Highly Active” OR “Anti-Retroviral Agents” OR antiretroviral OR art OR medication))
