# Supplementary material for: Oral Health Status of Children and Adolescents Living with HIV Undergoing Antiretroviral Therapy: A Systematic Review and Meta-Analysis
Source: Int J Environ Res Public Health. 2022 Oct 8;19(19):12864. doi: 10.3390/ijerph191912864 (PMC9564723; doi:10.3390/ijerph191912864)
Supplement: Supplementary file 1 [file ijerph-19-12864-s001.zip › ijerph-1892954-supplementary.pdf]

Table S1. GRADE summary of findings table for the primary outcome (caries prevention and arrest) and secondary outcome.

Outcome	Comparison	*No. of studies*	*No. of HIV-infected and healthy controls*	*Results*	Risk of bias[a]	Inconsistency[b] *I² statistics*	*Heterogenicity χ²test (p value)*	Indirectness[c]	Imprecision[d]	Publication bias[e]	Quality of evidence (GRADE)
**HIV-infected individuals under antiretroviral medications versus healthy controls**											
**Dental caries experience**	Prevalence	2	515 & 513	Inconsistent results	Not serious	**86.7%***	**0.006****	Not serious	Not serious	N/A	⊕OOO very low
					--	↓	↓	--	--	--	
**Periodontal condition**	Prevalence	2	125 &125	Significantly higher among HIV-infected children under antiretroviral medications	Not serious	**69.7%***	0.069	Not serious	**Serious**	N/A	⊕OOO very low
					--	↓	--	--	↓	--	
**Angular cheilitis**	Prevalence	2	112 &112	No significant difference	Not serious	0.0%	0.667	Not serious	**Serious**	N/A	⊕⊕OO low
					--	--	--	--	↓	--	
**Linear gingival erythema**	Prevalence	2	112 &112	No significant difference	Not serious	0.0%	1.00	Not serious	**Serious**	N/A	⊕⊕OO low
					--	--	--	--	↓	--	
**Recurrent oral ulceration**	Prevalence	3	142 &142	Significantly higher among HIV-infected children under antiretroviral medications	Not serious	0.0%	0.784	Not serious	**Serious**	N/A	⊕⊕OO low
					--	--	--	--	↓	--	
**Oral candidiasis**	Prevalence	5	216 & 262	Significantly higher among HIV-infected children under antiretroviral medications	Not serious	0.0%	0.944	Not serious	**Serious**	N/A	⊕⊕OO low
					--	--	--	--	↓	--	
**Oral hairy leukoplakia**	Prevalence	2	112 &105	No significant difference	Not serious	0.0%	1.00	Not serious	**Serious**	N/A	⊕⊕OO low
					--	--	--	--	↓	--	
**Hyperpigmentation**	Prevalence	3	207 & 200	Significantly higher among	Not serious	0.0%	0.784	Not serious	**Serious**	N/A	⊕⊕OO low

	HIV-infected children under antiretroviral medications	--	--	--	--	↓	--

Legend of table: [a]Risk of bias: Considered as serious if overall half of the studies included were of serious risk of overall bias. [b]Inconsistency: Considered as serious when I^2 statistics ≥50% (*) and p-value of χ^2 test <0.05 (**). [c]Indirectness: Considered as serious when the applicability of findings were restricted in terms of population, intervention, comparator, and outcomes. [d]Imprecision: Considered as serious when the total number of events was below 300 for dichotomous outcomes or 400 for continuous outcomes ([#]), or when the upper and lower limits of 95% CI include both meaningful benefits and harm. [e]Publications bias: Considered as serious if p-value of Begg's funnel plot <0.05. Not applicable (N/A) if funnel plot could not be constricted given the limited number of studies. Publication bias was difficult to detect and thus no downgrading was performed. ↓: Downgrade by 1 level in quality of evidence. --: No change in quality of evidence.
